# Toward Shared Working Space of Human and Robotic Agents Through Dipole Flow Field for Dependable Path Planning

**DOI:** 10.3389/fnbot.2018.00028

**Published:** 2018-06-06

**Authors:** Lan Anh Trinh, Mikael Ekström, Baran Cürüklü

**Affiliations:** School of Innovation, Design, and Technology, Mälardalen University, Västerås, Sweden

**Keywords:** navigation field, Theta star algorithm, dependability, multiple agents, path planning, dynamic environment

## Abstract

Recent industrial developments in autonomous systems, or agents, which assume that humans and the agents share the same space or even work in close proximity, open for new challenges in robotics, especially in motion planning and control. In these settings, the control system should be able to provide these agents a reliable path following control when they are working in a group or in collaboration with one or several humans in complex and dynamic environments. In such scenarios, these agents are not only moving to reach their goals, i.e., locations, they are also aware of the movements of other entities to find a collision-free path. Thus, this paper proposes a dependable, i.e., safe, reliable and effective, path planning algorithm for a group of agents that share their working space with humans. Firstly, the method employs the Theta^*^ algorithm to initialize the paths from a starting point to a goal for a set of agents. As Theta^*^ algorithm is computationally heavy, it only reruns when there is a significant change of the environment. To deal with the movements of the agents, a static flow field along the configured path is defined. This field is used by the agents to navigate and reach their goals even if the planned trajectories are changed. Secondly, a dipole field is calculated to avoid the collision of agents with other agents and human subjects. In this approach, each agent is assumed to be a source of a magnetic dipole field in which the magnetic moment is aligned with the moving direction of the agent. The magnetic dipole-dipole interactions between these agents generate repulsive forces to help them to avoid collision. The effectiveness of the proposed approach has been evaluated with extensive simulations. The results show that the static flow field is able to drive agents to the goals with a small number of requirements to update the path of agents. Meanwhile, the dipole flow field plays an important role to prevent collisions. The combination of these two fields results in a safe path planning algorithm, with a deterministic outcome, to navigate agents to their desired goals.

## 1. Introduction

Until recently, robots have played a critical role in the manufacturing industry where the automatic robots perform repetitive and sometimes heavy tasks. The majority of these solutions assume high precision with respect to movements and positioning of the robots, without relying on sensors, or at least extensive sensor feedback. However, technological advancements in recent years have resulted in a shift of attention from pre-programmed automatic solutions to (semi)-autonomous systems that can operate in unstructured environments, and even co-exist with humans. As a result of this shift, robots will be more involved in our daily activities. Thus, they will be allowed to have more interactions with humans, share working space with humans as well as make their own decisions with some accepted levels of uncertain information collected from the surrounding environment. For instance, there is a rise of interest in self-driving cars where the fully autonomous mode has been investigated to help drive the car in city centers, substandard roads or busy highways without causing accidents. In the health care domain, robots are assumed to assist elderly people in their daily activities. In this context, different levels of safety need to be taken into account, e.g., develop an autonomous control to avoid executing any movements that the users do not expect and also to prevent accident caused by a person being hit by the robot. The challenges, and the opportunities, in the health care domain becomes more evident considering care at home. Going back to the main application domain, i.e., industrial robotics, it is evident that the next generation solutions assume high degree of interaction and collaboration between mixed teams of humans and robots. Obviously, the approach taken by these solutions will not exclude today's standard solutions. Thus, it is most likely that different solutions will exist side by side in the near future.

Nevertheless, the developments in autonomous robots that co-existence of humans and robots, have opened new challenges in research areas of robotics, e.g., in motion planning and control. In particular, the control system should be able to provide the robots a reliable motion planning and control ability when the robots are working in a group or in collaboration with one or several humans in complex and dynamic environments. This means that the robots must meet certain requirements on trustworthiness/dependability in order to be allowed to work with humans. The dependability of a robotic agent is presented by main attributes including availability, i.e., the continuous operations of the system over a time interval, reliability, i.e., the ability of the system to provide correct services, and safety, i.e., the robotic agent must ensure safe controls to avoid any catastrophic consequences on users, other robots, and finally the environment. In order to implement a dependable robotic agent, important efforts have been attempted in several directions. Firstly, level of robot autonomy is automatically adaptive to the working context in order to address alternative complexities of environments. Secondly, the robot is willing to share the control with humans and other robots to optimize the working performance as well as to deal with complicated tasks that the robot cannot complete by itself. Lastly, to some extent, the robot must be able to handle the dynamic changes that occur in the environment, and to operate in accordance with the presence of other robots and humans in the same working space. This work mainly focuses on the last approach to enhance the robustness and dependability of the agents while working together with others and humans to complete a task.

Note also that, the high-level specification of a complicated movement of robots can be constructed through a sequence of lower level motion and path planning. A common problem is the movement of a robot arm, which can be composed of a sequence of trajectory planning and collision detection steps (Rubio et al., 2012, 2018). Therefore, motion and path planning are concerned as the basic, and separate, constructions for plans of robotic actions. Path planning is the process which is utilized to construct a collision-free path from a starting point to a destination given a full, partial or dynamic map. Motion planning, meanwhile, is the progress in which a series of actions are needed to be defined to follow the planned path. The most common practice in robotics is to address the navigation problem using path planning, i.e., pure geometric planning from point to point, then motion planning is to realize the feasibility of the path. As the output of path planning will later determine the way to plan robots' motion, the path planning algorithm is better incorporated with motion planning to optimize the movement of the robot. This means that the path planning could be realized at every locations within the form of navigation field to make transformation from path to motion planning easier. Besides, the moving path must be estimated to avoid many changes of moving directions to save energy used to perform movements.

With regard to above mentioned issues, this paper addresses path planning of robotic agents in the context of shared working space of humans and agents. The aim is to develop a path planning algorithm to deal with the dynamic changes of environments and complicated maps with multiple static obstacles having a wide range of shapes. The algorithm also helps agents avoid collisions with humans and others in the shared environment, in which a group of agents are designed to collaborate with each in order to plan their optimal paths, in real-time. Finally, how to combine the aforementioned factors of motion planning into the developed path planning algorithm is investigated.

So far, numerous path planning approaches have been proposed to address control movement of robots. Most of them have been focused on searching to find a path from a starting point to a destination in either static or dynamic map. Meanwhile, a family of path planning algorithms address the problem of avoiding moving obstacles with field-based approaches. Regarding search-based algorithms, one of the most conventional yet still effective approaches for the navigation of an agent in a large map is related to Dijkstra and its extension of A^*^ searching algorithm (Cormen et al., 2009; Yershov and LaValle, 2011), and incremental search (Koenig et al., 2004b). In detail, the A^*^ algorithm improves the Dijkstra's algorithm by approximating the cost-to-go function with heuristic knowledge to reduce the searching space to the goal. Meanwhile, incremental search algorithms seek for the shortest paths by utilizing the results of similar searches to make the search faster, instead of solving each search problem separately. By applying incremental search on top of the A^*^, Koenig et al. (2004a) developed lifelong planning A^*^ (LPA^*^) as an initial variant of A^*^, in order to address path planning for dynamic graphs with changing edge costs. In the D^*^ algorithm (Stentz, 1994), incremental search is applied to repeatedly update the shortest paths between the current position of a robot and a goal, during the robot's approach to the goal. Koenig and Likhachev (2005) improved the D^*^ by LPA^*^ and alternatively Sun et al. (2009) developed dynamic fringe saving A^*^ to reuse the OPEN and CLOSED lists from previous A^*^ searches. Although different variants of A^*^ are able to address a graph change due to the moving of a robot to a new vertex, or the updates of edge costs, those algorithms still face difficulties to deal with moving obstacles. In addition, as stated by Hu and Brady (1997), a probabilistic approach is necessary to model the uncertainties of mobile obstacles in the environment. However, the complexity of path planning will be significantly increased if either the cost of the edges, or the links of the graph are presented by random variables.

In order to handle the uncertainties of observed obstacles, a field-based approach is another way to find the path for the agents. The field is calculated for each location, in time and space, and determines the directions of movement of an agent to reach the destination. The field consists of a repulsive field to push the agent away from the obstacles, and an attractive field to pull the agent toward the goal. For instance, Ok et al. (2013) proposed Voronoi uncertainty field which is build from Voronoi diagram from the start to the goal to create the attractive field and the repulsive field from the robot to the obstacles. The works of Wang and Chirikjian (2000) and later Golan et al. (2017) presented an artificial potential field based on the exchanges of heat flow. If obstacles are visualized as hot objects, the target is then presented as the cold one and the temperature is discretized at each location on the grid. The temperature gradient solved by partial differential equation generates the appropriate forces to drive the robot. One of the big issues of using the potential field is that the repulsive field may push the agent to reach other obstacles or statures with the attractive field. Due to these problems, the agent may be trapped into a local optimum or loose its way toward the goal. To mitigate the local converge to a local optimal, some additions to the potential field have been introduced. Valbuena and Tanner (2012) proposed the way of adding velocity constraints, meanwhile García-Delgado et al. (2015) extended the repulsive function with the change of magnitude dependent on the angle between the attractive force and the obstacle. The main aim is to avoid the cancellation of the repulsive and attractive forces when applied in opposite orientations. However, the interactions of the agents with the environment, especially changes in the map, were not clearly addressed in above mentioned works. Besides, most of field-based navigation approaches lack the global information of a feasible path to the destination that could actually help avoid a trap that would lead to a local optimum.

Controlling the speed and directions of a robot are also key factors, which plays a role to provide the robot a collision free path. Owen and Montano (2005, 2006) defined velocity space to estimate the arrival time of moving objects to a region of potential collisions and thereby potential solutions to avoid these collisions. The velocity space in which the motion of the robot, as well as static and moving objects are mapped, is applied to predict when the collision may happen and when the robot may escape from the collision. Damas and Santos-Victor (2009) developed a map of forbidden velocity zones which is constructed as a limit on the velocity of the robot to avoid collision with obstacles. When the robot moves into the forbidden zones, it may adjust its speed to avoid the obstacles. Berg et al. (2008), Wilkie et al. (2009), and Berg et al. (2011) further integrated the acceleration while Lee et al. (2017) concerned the shape of the robots as an ellipse for obstacle avoidance. Yoo and Kim (2010) proposed a modified uni-vector field to present obstacles with respect to relative their velocities and positions where the gaze control which concerned the error of localization and the distances to surrounding obstacles was also combined into the system to find the best moving trajectory. Belkhouche. (2009) introduced virtual plane to present moving objects with information of velocity into stationary ones. As a consequence, path finding in a dynamic environments is converted to a simpler problem of navigation in a static environment. However, it is noted that, it is not always optimal to use velocity planning when to drive the robot. Using only velocity control for path planning usually results in oscillatory motion. Given a typical differential drive mobile robot, there are a number of constraints on the linear and angular velocities, as well as the acceleration, in order to save energy for extending operation time, and finding the path to the goals with few turns. To the best of our knowledge, these concerns have not been investigated extensively in combination with obstacle avoidance in dynamic environment.

In order to address the above mentioned issues, in this paper, a novel method for path planning of mobile agents, in the shared working environment of human and agents, called as the dipole flow field, is proposed. The dipole flow field combines both global and local path planning in a unique framework. For global planning, the method applies any-angle path planning algorithm of Theta^*^ (Nash et al., 2010) to generate smooth paths with few turns, from a starting point to a goal for a pool of agents. Although different A^*^ variants of any-angle path planning haven been proposed, such as A^*^ post-smoothing, block A^*^ (Yap et al., 2011) and field D^*^ (Ferguson and Stentz, 2006), the Theta^*^ is able to provide the most optimal path with simple and effective implementation (Uras and Koenig, 2015). As the computations of the Theta^*^ algorithm is costly for a big map, the algorithm is updated when there is a significant change on the static map of the environment. To cope with dynamic movements of the agents, a static flow field along the planned path is defined to attract the agent back to continue reaching the goal even when the agents may be deviated from the planned path. In addition, a dipole field is used to avoid the collision of the agents with others and human within shared working space. To the best of our knowledge, most conventional approaches attempt to generate the pushing forces based only on the location of the agents, whereas in this work, it is assumed that, those should be better aligned with both moving directions and velocity magnitudes of different agents. The generated dipole field is able to push other agents far away based on their respective moving directions and the velocity magnitude of the agents. Static flow field and dipole field are combined to assure a dependable path of each agent from the starting point to the goal without colliding with each other.

The rest of paper is organized as follows. The methodology of the proposed path planning based on dipole flow field is presented in section 2. The evaluation of the proposed method through the experimental results is described in section 3. Finally, the paper is concluded with discussion in section 4.

## 2. Methodology

In this section the agent architecture together with the different modules used for path planning are described in section 2.1. Meanwhile the core path planning algorithm is presented in section 2.2.

### 2.1. Autonomous agent architecture

**Figure 1:**
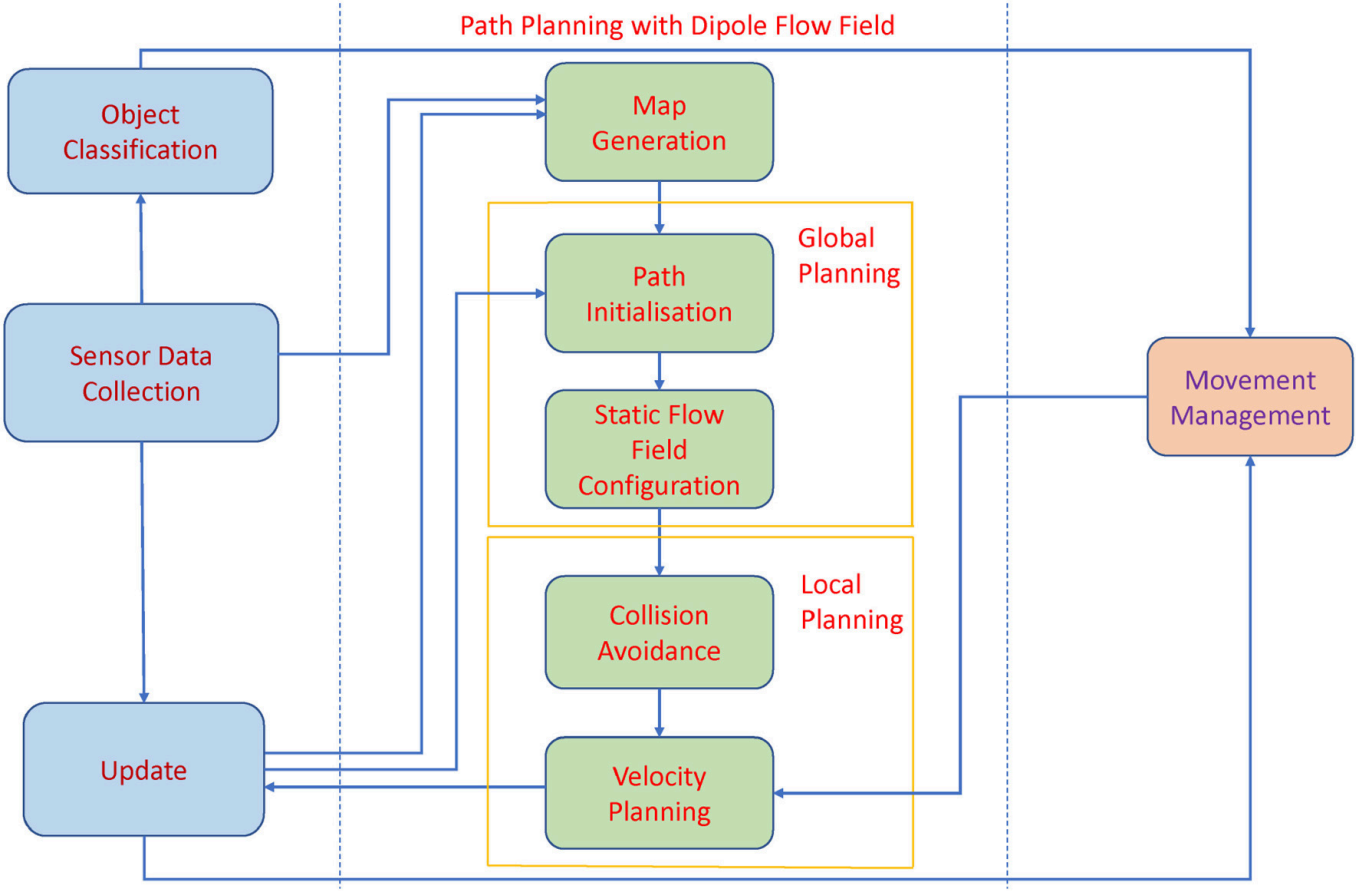
The Architecture of Autonomous agent. The backbone of the path planning algorithm consists of the *Map Generation* module, *Global Planning* including the *Path Initialization* module and the *Static Flow Field Configuration* module, and *Local Planning* including the *Collision Avoidance* module and the *Velocity Planning* module.

The overall architecture of the autonomous agent to support the proposed planning algorithm is presented in Figure 1. The core algorithm includes the following five modules, *Map Generation, Path Initialization, Static Flow Field Configuration, Collision Avoidance* and *Velocity Planning*. In addition, there are four external modules including *Sensor Data Collection, Update, Object Classification*, and *Movement Management* to help the planning algorithm collect information from the surrounding environment and update control.

#### 2.1.1. Path planning architecture

After that the global information of the environment is acquired from the external modules, a 2-D map is generated. The 2-D map is presented as a binary map in which static objects and obstacles are shown as black areas whereas the allowed moving areas are illustrated with white color. Global path planning with Theta^*^ algorithm is applied in the *Path Initialization* module to initiate a path from the starting point to the destination for the agents. While moving to the goal, the agent may deviate from the original path due to obstacle avoidance, or accumulated errors related to velocity and pose estimating. As a result, a static flow field generated in the *Static Flow Field Configuration* module will drive the agent back to the designed path. Only when the agent moves far away from the region covered by the static flow field, Theta^*^ is activated to renew the path from the current position to the goal. After the static flow field is configured, the agents start moving to reach their individual goals, while checking for collision with other moving objects. The dipole field is calculated in the *Collision Avoidance* module to avoid collision with the agents. Finally, the motions of the agents are controlled by the superposition of the static flow field, and dynamic dipole field to generate the dipole-flow force. The dipole-flow force is presented by the adjustments of the agents' heading angles. A velocity function is established to help the agent well adapt its moving velocity according to two factors, energy consumption and obstacle avoidance. If there is no collision, the agent will move with a stable speed along the configured path. Meanwhile, if there is a dynamic obstacle, the agent needs to adjust its moving direction to avoid the obstacle while still maintaining, or at least minimizing, the deviation from the time to goal.

#### 2.1.2. External modules to support path planning algorithm

The *Sensor Data Collection* module is designed to continuously collect information of the environment. For instance, the visual data obtained from a camera, together with the data from the other sensors, is used to build the map of the environment and to recognize different objects. The pose of the robot is collected from the inertial measurement unit (IMU). Similarly, the positioning tracking system registers the position of the robot within the map. The *Object Classification* module receives the data from the *Sensor Data Collection* module to determine which objects in the environment that are static objects and which ones are moving objects. In this work, the proposed path planning algorithm deals with two types of moving objects. Firstly, autonomous agents, which share information about their locations, and velocities, with the other agents. Secondly, uncontrolled moving object, e.g., a human, who can suddenly appear in the working space of the agents. Especially when the human subjects are present, the agents need to adjust their movement to avoid them. The *Movement Management* module plays a central role in managing the location, and moving trajectories of all agents and human(s) found in the environment. The data from the *Movement Management* module is sent to the path planning algorithm for velocity estimation. The *Update* module updates the internal model based on the changes in the environment, and applies the control commands from the *Velocity Planning* module to move the agents accordingly.

### 2.2. Path planning with dipole flow field

In this section, the dipole flow field is firstly formulated by the combination of the static flow field and the dynamic dipole field. Later, the direction of the dipole flow field at every point is turned into velocity planning to control the linear and angular velocities of agents.

#### 2.2.1. Static flow field for global path planning

The global path consists of a sequence of line segments from the start to the goal, and is configured using the Theta^*^ algorithm. Within the neighborhood of the found path, a navigation field parallel to the path segment, is defined, as the static flow field.

##### 2.2.1.1. Path initialization

To initialize the path from a starting point to an ending point, the Theta^*^ algorithm is applied. This algorithm improves A^*^ by adding a line-of-sight (LOS) detection to each search iteration to find a less zigzaggy path than those generated by A^*^ and its other variants. The primary difference between the Theta^*^ and the others is that the Theta^*^ algorithm determines the parent of a node to be any node in the searching space. Thence, the LOS detection feature is purposed to help decrease the undesired expanding by checking for whether the offspring node and the parent are in a straight line, i.e., line-of-sight. By this means, the path found by Theta^*^ is not a connection of adjacent nodes but a connection of line-of-sight ones. The pseudo codes of the Theta^*^ is described in Algorithm 1.

**Algorithm 1:**
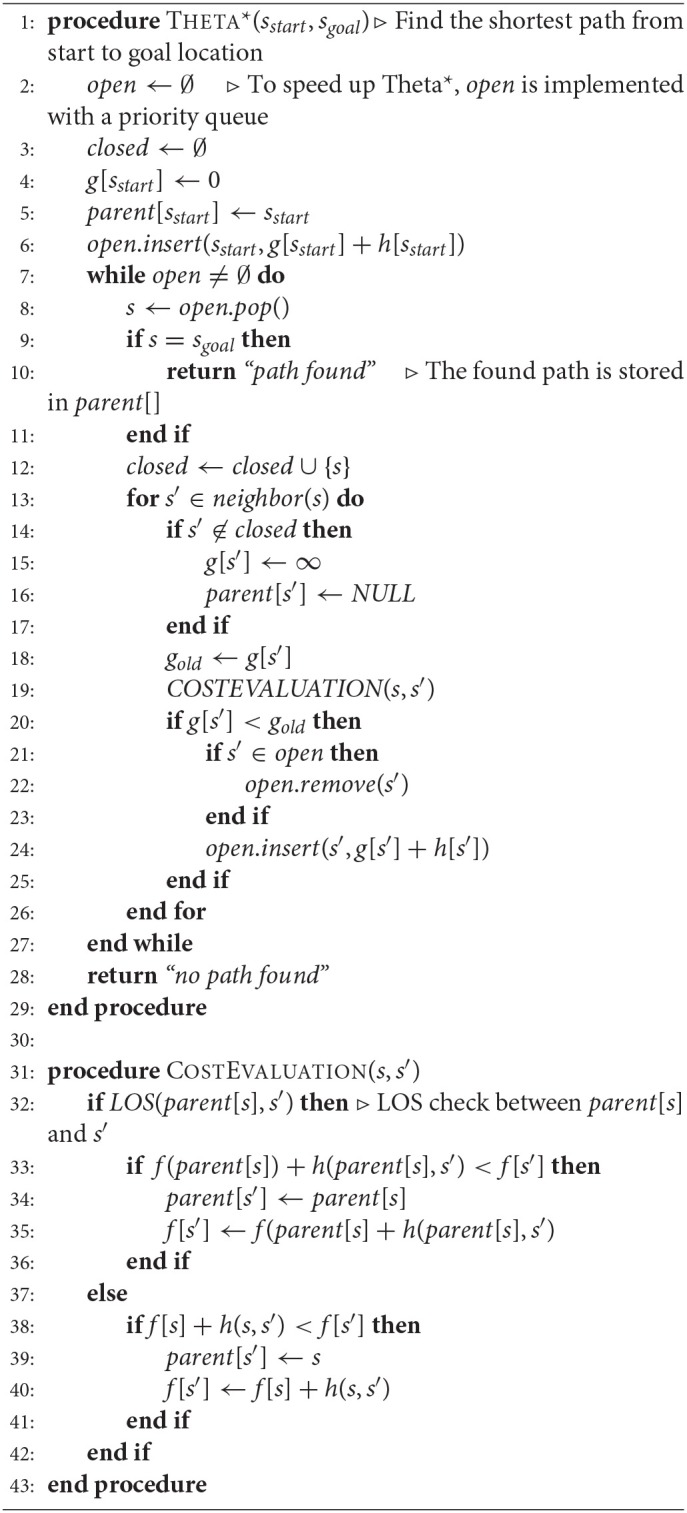
Theta^*^ algorithm

As a heuristic-based search algorithm, Theta^*^ approximates the length of the shortest path based on cost evaluation. The cost evaluation is conducted from the *f*-value, i.e., the lowest cost from the starting node to the last node, *s*, in a path, referred to as *f*(*s*), and a heuristic value called *h*-value which is the cost estimation from the node *s* the goal. The estimated cost of the cheapest node through node *s* is, thus expressed by:

(1) $$f(s^{\prime })=f(s)+h(s,s^{\prime }).$$

In this work, the heuristic function is simply defined as the Euclidean distance i.e., *h*(*s*, *s′*) = *w.Euclidean*(*s*, *s′*) where *w* is a weight that determines the size of the area to search for the optimal path around the straight-line between *s* and *s*′. With *w* > 1, Theta^*^ is able to reduce the searching area but may return a longer path, therefore the value *w* = 1 is used in this work to search for the shortest path. It is assumed that the straight line distance between two nodes would be never longer than any other path connecting them. However, the shortest path generated by the A^*^ algorithm is connected by the neighboring grid nodes, and thus entails many turning points to the robot. The path found by Theta^*^ is a sequence of LOS nodes so that it is smoother with few turns and closer to a straight-line path between the start and the goal. The algorithm for LOS function is implemented with a drawing-line algorithm in graphics to optimize processing time and is referred to approach proposed by Nash et al. (2010).

**Figure 2:**
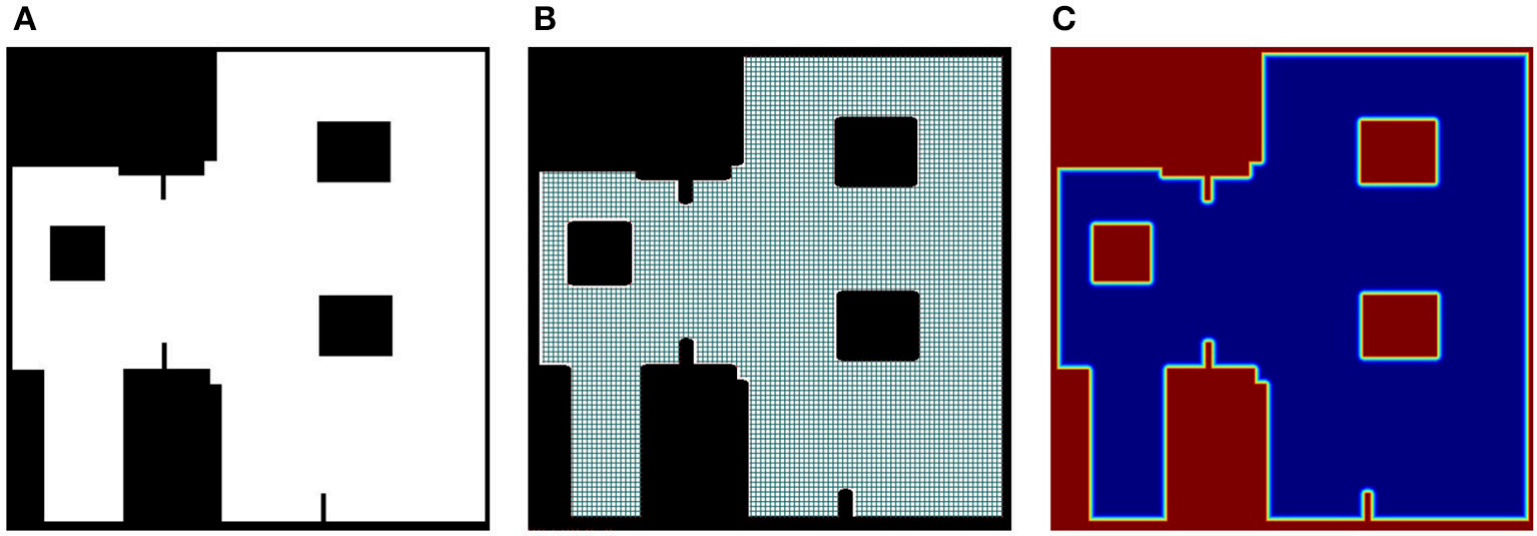
Binary map for static flow field and derived information, **(A)** the original binary map in which the white pixel presents available regions of agents, **(B)** the grid-based graph derived from the binary map, and **(C)** the corresponding repulsive field in which the amplitude of the field from the lowest to the highest is mapped into colors from blue to red respectively.

As mentioned in section 2.1.1, the input to the Theta^*^ algorithm is the binary map of the environment (Figure 2A). However, to avoid searching the path on a dense graph, a grid-based graph is used (as visualized in Figure 2B). The obstacle areas are also dilated corresponding to the size of agents so that the path found by Theta^*^ will not cause the boundary of the agent touching the edges of the map while the agent is moving.

##### 2.2.1.2. Path configuration with static flow field

**Figure 3:**
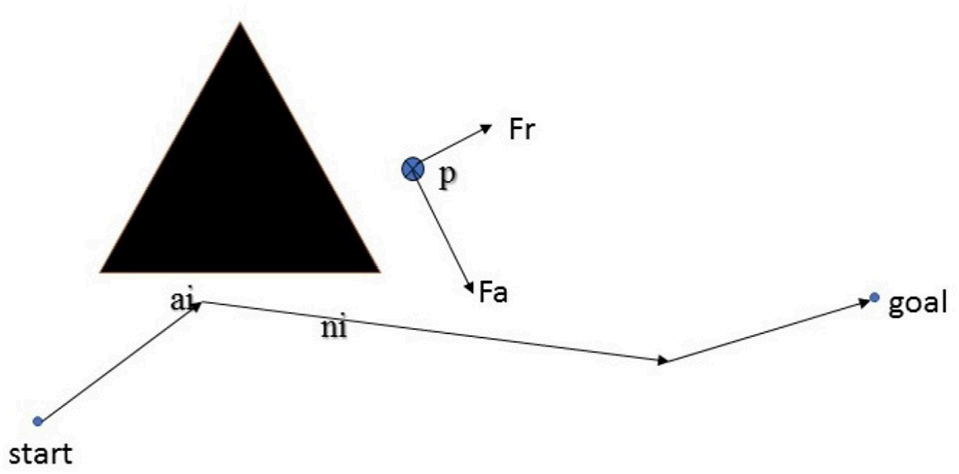
The configuration of the global path.

Searching for a global path from a start to a goal in a big map is a computationally heavy task, thus it is not desired to re-calculate the path for small updates of the map, or small deviations from the configured path. The static flow field is to draw the agents back to their moving paths in those situations. In the form of force interaction, the static flow field is also easily combined with a dynamic field for obstacle avoidance. As the shortest path found by Theta^*^ is the connection of several line segments, the static flow field is created within the neighbor of the line segments. For each path, it is assumed that there are *n* line segments from the start to the end points. Each line segment *i* is presented in a vector form of **x**(*t*) = **a**_*i*_ + *t***n**_*i*_ where **a**_*i*_ is the starting of the line segment and **n**_*i*_ is the unit vector of the line. To ensure that the static flow field will draw the agent to its goal, those line segments also include the last line segment with **a**_*i*_ is set to the goal and **n**_*i*_ to a zero vector. The flow field force at the point **p** close to the provided path found by Theta^*^ is calculated by

(2) $$F_{flow}(\text{p})=F_{a}(\text{p})+F_{r}(\text{p})$$

where *F*_*a*_(**p**) is the attractive force to draw an agent back to the configured path, and *F*_*r*_(**p**) is the repulsive force from nearby static obstacles. The configuration of the global path and the formulation of the flow force are described in Figure 3.

**Figure 4:**
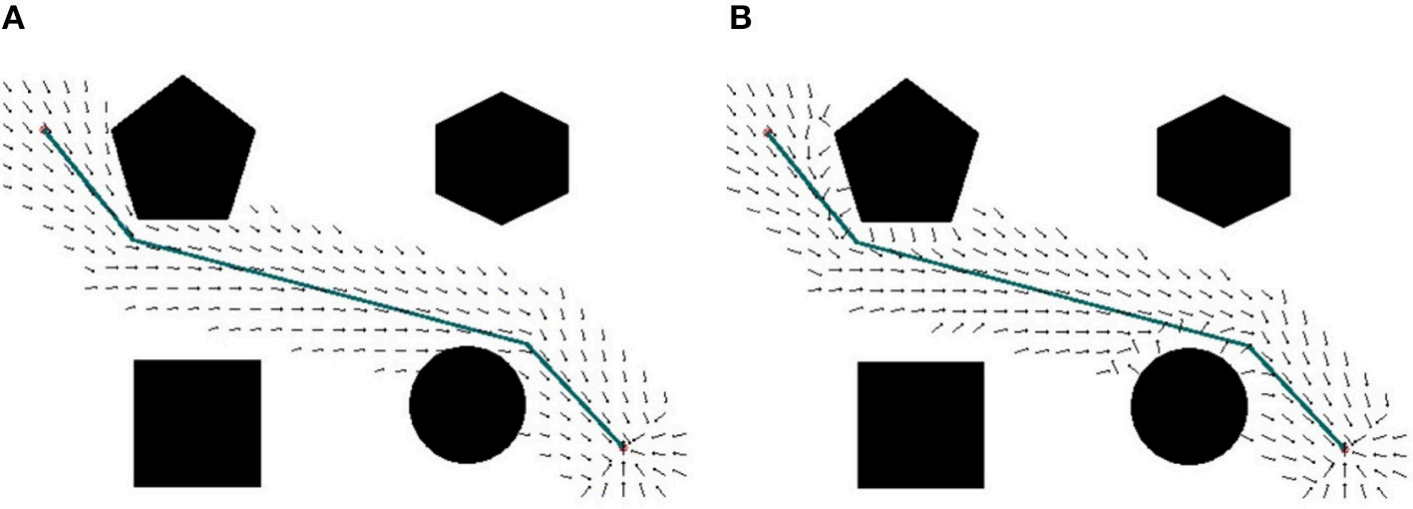
The representation of the static flow field (unity vectors), **(A)** the initial path with the configured static attractive field, **(B)** the static flow field with added repulsive force to the obstacles.

Let *F*_*a*_*i*__(**p**) be the attractive force of a point to each line segment and expressed by:

(3) $$F_{a_{i}}(\text{p})=\left(1-{\text{e}}^{-k_{1}d\left(\text{p},{\text{a}}_{i}\right)}\right)\left(\left({\text{a}}_{i}-\text{p}\right)-(\left({\text{a}}_{i}-\text{p}\right)\cdot {\text{n}}_{i}){\text{n}}_{i}\right)+k_{2}{\text{e}}^{-k_{1}d\left(\text{p},{\text{a}}_{i}\right)}{\text{n}}_{i}$$

where *d*(**p**, **a**_*i*_) is the distance from the point **p** to the line segment *i*-th, *k*_1_, *k*_2_ are constants, and “ · ” denotes the inner product of two vectors. As **n**_*i*_ is a unit vector, the vector (**a**_*i*_ − **p**) − ((**a**_*i*_ − **p**)·**n**_*i*_)**n**_*i*_ is normalized before Equation (3) is calculated. Two constants *k*_1_ = 0.01 and *k*_2_ = 1 are selected to control the impact of the first and second terms of Equation (3). The attractive force *F*_*a*_(**p**) is set equal to the attractive force $F_{{\text{a}}_{i}^{*}}$ of the line segment ${\text{a}}_{i}^{*}$ closest to the point **p**, ${\text{a}}_{i}^{*}=\underset{{\text{a}}_{i}}{\mathrm{argmin}}d\left(\text{p},{\text{a}}_{i}\right)$. Meanwhile, the repulsive force *F*_*r*_(**p**) = −∇*U*_*rep*_(**p**) is the negative gradient of the repulsive field:

(4) $$U_{rep}(\text{p})=\{\begin{array}{cc} \eta {\left(\frac{1}{d(\text{p},{\text{p}}_{0})}-\frac{1}{d_{0}}\right)}^{2} & d(\text{p},{\text{p}}_{0})\leq d_{0}\\ 0 & d(\text{p},{\text{p}}_{0})>d_{0} \end{array}$$

in which *d*(**p**, **p**_0_) = ||**p** − **p**_0_|| is the Euclidean distance from the agent's position **p** to the closest obstacle's position **p**_0_, *d*_0_ is the influence distance of the force, and η is a positive constant. To avoid singularities of Equation (4) when *d*(**p**, **p**_0_) = 0, a linear transformation *f*(*d*) = κ*d* + 1 is applied to map *d*(**p**, **p**_0_) and *d*_0_ to non-zero values *f*(*d*(**p**, **p**_0_)) and *f*(*d*_0_) in Equation (4). The influence distance of the force, *d*_0_, is selected based on the size of agents (the diameter of agents) to prevent touching the agents to static obstacles. With respect to 0 < *d*_0_ < 100, κ = 0.01 and η = 10^4^ are chosen. An example of repulsive field of the binary map given in Figures 2A,B is shown in Figure 2C. The static flow fields without and with added repulsive forces are presented in Figures 4A,B respectively.

The affecting area of the static flow field is determined by the window size (*W*). This means that the static flow field remains influence on the agent if the distance from the agent to the designed path is less than *W*. Once the agent moves out of the affecting area, the Theta^*^ algorithm needs to be recalculated to update a new static flow field.

#### 2.2.2. Dynamic dipole field

To cope with the problem of collision avoidance, the dipole field for each dynamic object is generated. The development of the dipole field is inspired by the way that humans naturally avoid moving obstacles: When facing an obstacle that is approaching, the human may turn, and continue to move, to avoid the obstacle instead of going backwards. Such a movement shows a moving trajectory similar to that of a dipole magnetic field line. This method is also a more skillful obstacle avoidance strategy than the conventional method of using radial potential field. Munasinghe et al. (2005) introduced an implementation of this obstacle avoidance method by designing a force to drive a robot through an elliptical trajectory to go around and then behind obstacles. In the work of Igarashi et al. (2010) the dipole characteristics is expressed as a vector field to push an object to a goal. In this work, to model the moving behavior of agents, instead of developing dipole-like vector field the theory of dipole magnetic field in physics is directly applied. Each agent can be seen as a source of a magnetic dipole field, in which the magnetic moment is proportional to the velocity vector of the agent. This means that the orientation of the moment is aligned with the moving direction of the agent and the magnitude of the moment is equal to the speed of the agent. The aim of having the moment proportional to the speed is to ensure that among different obstacles having the same distance to the agent, the one with the larger speed will contribute a stronger effect on driving the agent.

In physics, the magnetic field *M* of the dipole moment vector *m* is expressed by:

(5) $$\text{M}(\text{m},\text{d})=\rho (3(\text{m}\cdot \hat{\text{d}})\hat{\text{d}}-\text{m})/d^{3}$$

where **d** is the distance vector, *d* = ||**d**|| is distance between two agents, and $\hat{d}=\text{d}/||\text{d}||$ is a unit vector. The magnetic constant ρ = 1/3 (3ρ = 1) is applied in this work instead of using $\rho =\frac{\mu_{0}}{4\pi }$ in electromagnetic theory (μ_0_ is the permeability of free space). An agent with the magnetic moment **m_j_** within the magnetic field **M_k_** generated by the other magnetic source **m_k_** would be affected by the force:

(6) $$\text{F}=\nabla {\text{m}}_{\text{j}}\cdot {\text{M}}_{\text{k}}$$

where the gradient ∇ presents the changes of the quantity **m_j_** · **M_k_** per unit distance. Hereby, the repulsive force of an agent *k* on an agent *j* can be formulated by:

(7) $$\begin{array}{l} {\text{F}}_{dipole}({\text{m}}_{j},{\text{m}}_{k},\text{d})=\rho \nabla \left({\text{m}}_{\text{j}}\cdot \frac{3({\text{m}}_{\text{k}}\cdot \hat{\text{d}})\hat{\text{d}}-{\text{m}}_{\text{k}}}{d^{3}}\right)\\ \;=\rho \nabla \frac{3({\text{m}}_{\text{j}}\cdot \text{d})({\text{m}}_{\text{k}}\cdot \text{d})}{d^{5}}-\frac{\left({\text{m}}_{\text{j}}\cdot {\text{m}}_{\text{k}}\right)}{d^{3}}\\ \;=\rho (3({\text{m}}_{\text{j}}\cdot \text{d})({\text{m}}_{\text{k}}\cdot \text{d})\nabla \frac{1}{d^{5}}+\frac{3({\text{m}}_{\text{j}}\cdot \text{d})}{d^{5}}\nabla ({\text{m}}_{\text{k}}\cdot \text{d})\\ \;+\frac{3({\text{m}}_{\text{k}}\cdot \text{d})}{d^{5}}\nabla ({\text{m}}_{\text{j}}\cdot \text{d})-({\text{m}}_{\text{j}}\cdot {\text{m}}_{\text{k}})\nabla \frac{1}{d^{3}}))\\ \;=\frac{3\rho }{d^{4}}(({\text{m}}_{j}\cdot \hat{d}){\text{m}}_{k}+({\text{m}}_{k}\cdot \hat{d}){\text{m}}_{j}\\ \;+({\text{m}}_{j}\cdot {\text{m}}_{k})\hat{d}-5({\text{m}}_{j}\cdot \hat{d})({\text{m}}_{k}\cdot \hat{d})\hat{d}) \end{array}$$

where **m**_*j*_, and **m**_*k*_ are the dipole moments of the agents. To lead to Equation (7), the gradients of two functions, $\nabla \frac{1}{d^{n}}=-n\frac{\text{d}}{d^{n+2}}$ and ∇(**m**·**d**) = **m**, are used.

The magnetic force **F**_*dipole*_(**m**_*j*_, **m**_*k*_, **d**) is aligned with the direction of from **m_k_** to **m_j_** to generate repulsive forces. This means **F**_*dipole*_(**m**_*j*_, **m**_*k*_, **d**) will be reversed (turn about 180 degrees around the origin at an agent) if it has an opposite direction of a vector pointing from an agent *k* to an agent *j*. In order to increase the interaction range of dipole field, an adjustment factor γ , 0 < γ ≤ 1 and close to one (γ ≈ 1), is added as follows:

(8) $$\begin{array}{l} {\text{F}}_{dipole}({\text{m}}_{j},{\text{m}}_{k},\text{d})=\frac{3\rho }{d^{4\gamma }}(({\text{m}}_{j}\cdot \hat{d}){\text{m}}_{k}+({\text{m}}_{k}\cdot \hat{d}){\text{m}}_{j}\\ \;+({\text{m}}_{j}\cdot {\text{m}}_{k})\hat{d}-5({\text{m}}_{j}\cdot \hat{d})({\text{m}}_{k}\cdot \hat{d})\hat{d}). \end{array}$$

The smaller value γ is, the further distance the dipole field of one agent has influence on the others. In addition, a small term ϵ = 10^−12^ is added into *d* in the denominator of Equation (8) to avoid singularities.

#### 2.2.3. Dipole flow field

An agent needs to adjust its moving path according to its relative locations and orientations to other agents. Also, the agent concerns the possible collisions with uncontrolled moving objects, i.e., humans, which does not share information about their locations, and intentions regarding how they will move. Assume that there are a set of *N* agents, i.e., robots A={j|j∈1,2,…,N} in the working space. All agents are designed using the same architecture to cooperate with each other to plan global movements so that each of them transmits location information to the other agents in A. Let $O_{j}=\left\{o_{j}|o_{j}\in 1,2,\ldots ,N_{j}\right\}$ be a set of *N*_*j*_ human subjects recognized by the agent *j* within its detecting range. In this context, the relative location and velocity information about human subjects are estimations from observations over time. The dipole flow field for an agent *j* is formulated by integration of the static flow field, and the dynamic dipole field as:

(9) $$\begin{array}{l} {\text{F}}_{df}^{\left(j\right)}=\alpha {\text{F}}_{flow}^{\left(j\right)}/\|{\text{F}}_{flow}^{\left(j\right)}\|+\beta_{A}\sum_{k\in A,k\neq j}{\text{F}}_{dipole}({\text{m}}_{j},{\text{m}}_{k},{\text{d}}_{jk})\\ \;+\beta_{O}\sum_{l\in O_{j}}{\text{F}}_{dipole}({\text{m}}_{j},{\text{m}}_{l},{\text{d}}_{jl}) \end{array}$$

where $||{\text{F}}_{flow}^{\left(j\right)}||=\sqrt{{\left({\text{F}}_{flow}^{{\left(j\right)}_{x}}\right)}^{2}+{\left({\text{F}}_{flow}^{{\left(j\right)}_{y}}\right)}^{2}}$ is the magnitude of the flow force ${\text{F}}_{flow}^{\left(j\right)}={\left[F_{flow}^{{\left(j\right)}_{x}},F_{flow}^{{\left(j\right)}_{y}}\right]}^{T}$, here α, β_*A*_, and β_*O*_ are constants. Those constants determine the impact of dipole flow forces over static flow forces to control the moving of agents. Since the static flow force is normalized in Equation (9), the coefficient α>0 represents for the magnitude of the static flow field term. To simply reflect the effective area of the static flow field, α = 10 is chosen (correspondent to the agents' diameter of 1*m*, or 10 pixels, in all experiments). Meanwhile, the dipole field coefficients, β_*A*_ and β_*O*_, determine the effecting area of the dipole field. It is able to define this area of influence of the agent (*k*) on (*j*) by a circle $C_{jk}$ that has a center at the agent (*k*) and a radius *r*_*jk*_ to ensure that if *d*_*jk*_ < *r*_*jk*_ then β_*A*_||**F**_*dipole*_(**m**_*j*_, **m**_*k*_, **d**_*jk*_)|| > α. As the magnitude of ||**F**_*dipole*_(**m**_*j*_, **m**_*k*_, **d**_*jk*_)|| is proportional to $1/d_{jk}^{4\gamma }$, the dipole forces have strong influence on the agent (*j*) when the agent is inside $C_{jk}$. On the contrary this influence is significantly decreased outside $C_{jk}$. In this work, the two constants β_*A*_ and β_*O*_ are set to be equal (β_*A*_ = β_*O*_) and defined to control the desired effective area of the dipole field. This area has a radius that is proportional to ${\left(\beta_{A}/\alpha \right)}^{-1/4\gamma }$ and to the magnitude of dipole moments of agents. It is also noted that two agents (*j*) and (*k*) receive the dipole forces with the same amplitude but with opposite directions. Only agents are affected by the dipole forces generated by human subjects. Thus, in the model human subjects are not subject to these forces.

#### 2.2.4. Velocity planning

**Figure 5:**
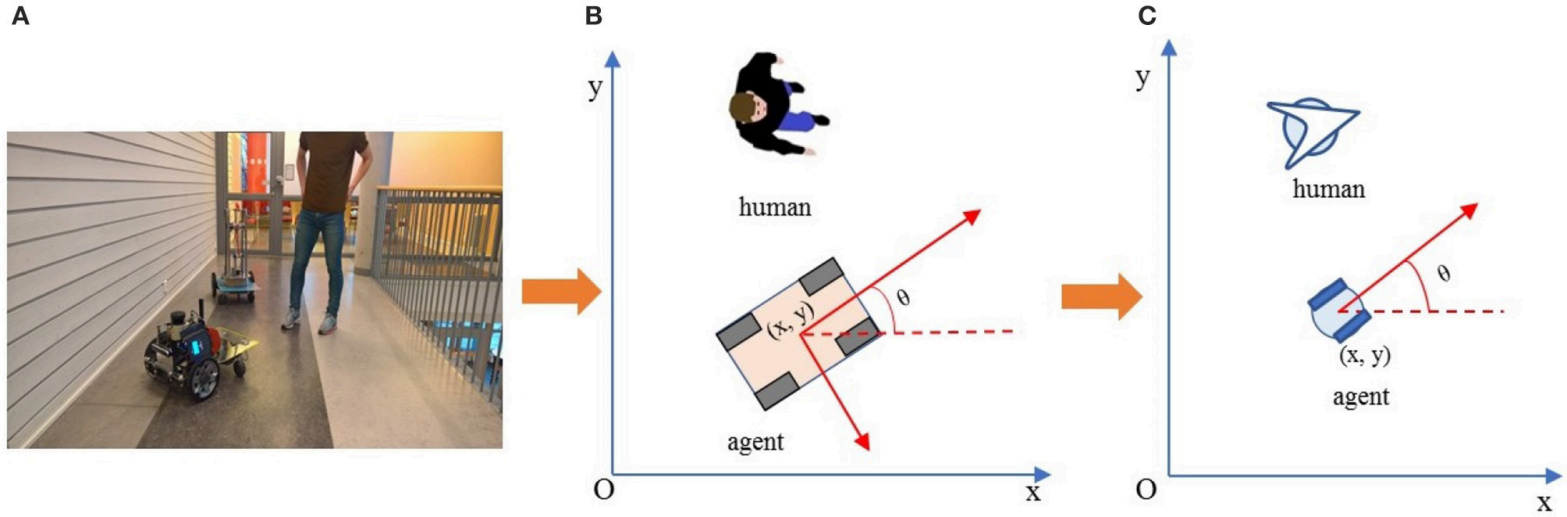
Visualization of an agent with kinematic parameters and human from **(A)** a real world space in **(B)** a 2D mapping space, and **(C)** a simplified visualization used in the proposed work.

In this work, an autonomous agent is presented by the kinematics model of a unicycle-type mobile robot (Morin and Samson, 2008). This model is chosen because despite its unicycle name, it approximates many widely used differential drive robots and can be easily extended to car-like mobile robots with two parallel driven wheels. The state of a robot (Figure 5) is described by a set of triple parameters **s**(*t*) = [*x*(*t*), *y*(*t*), θ(*t*)]^*T*^, and **r**(*t*) = [*x*(*t*), *y*(*t*)]^*T*^ are the coordinates, θ(*t*) is the orientation with respect to the *x*−axis of the robot, and *t* is time. The state *s*(*t*) is updated for every interval Δ*t* as:

(10) $$\begin{array}{l} x(t+\Delta t)=x(t)+u(t)\Delta t\cos\theta (t)\\ y(t+\Delta t)=y(t)+u(t)\Delta t\sin\theta (t)\\ \theta (t+\Delta t)=\theta (t)+\omega (t)\Delta t \end{array}$$

where *u*(*t*) and ω(*t*) are the linear and angular velocities of the agent respectively. Those velocities are computed by the following equation:

(11) $$\begin{array}{l} u(t)=k_{u}\tanh(||\text{r}(t)-{\text{r}}_{goal}||)\\ \omega (t)=-k_{\omega }\left(\theta (t)-\arctan\left(\frac{F_{df}^{y}}{F_{df}^{x}}\right)\right), \end{array}$$

where *k*_*u*_>0 and *k*_ω_>0 are two constant control gains. From this definition, the linear velocity *u*(*t*) is about *k*_*u*_ while an agent is moving on its ways and decays to zero when it is closer to the goal. Therefore, *k*_*u*_ is set to the expected speed of agent. Meanwhile, the angular velocity ω(*t*) is used to adjust the heading angle θ(*t*) of the agent to make the agent's orientation aligned with the direction of dipole field force **F**_*df*_. By this, the dipole flow field mainly affects the angular velocity ω(*t*) of the agent to drive it to the goal and to avoid the static obstacles, and moving objects when they are close. The second coefficient, *k*_ω_, controls how smooth the moving trajectory of the agent is and how fast the agent is able to adapt to the changes of the dipole field force.

## 3. Experiments

A number of experiments are conducted to validate the effectiveness of the proposed path planning algorithm. Different with most of existing approaches which have focused on alternative aspects of local or global path planning for a single agent, this work has developed a new promising framework to address the navigation problems of multiple agents sharing working space with human. This also adds a new dimension to existing solutions of robotics navigation with the definition of dynamic dipole field inspired from electromagnetic physics and of the static flow field based on Theta^*^ algorithm. Thus, the main aim of this section is to investigate on the characteristics of the proposed approach through various scenarios. The starting point is an experiment with static flow field. This experiment shows how this field is able to navigate agents to goals within the map of complicated static obstacles. The next experiment exploits the benefit of dipole field to help agents avoid moving obstacles coming from different directions. Finally, a set of experiments are conducted in order to evaluate how well the proposed path planning algorithm with the combination of flow and dipole flow fields, i.e., dipole-flow field, both drive agents toward the goals, and at the same time avoid collisions with moving objects. Data showing the agent-agent and human-agent distances in the presence of the dipole-flow field is also shown as part of the last experiment.

### 3.1. Static flow field

**Figure 6:**
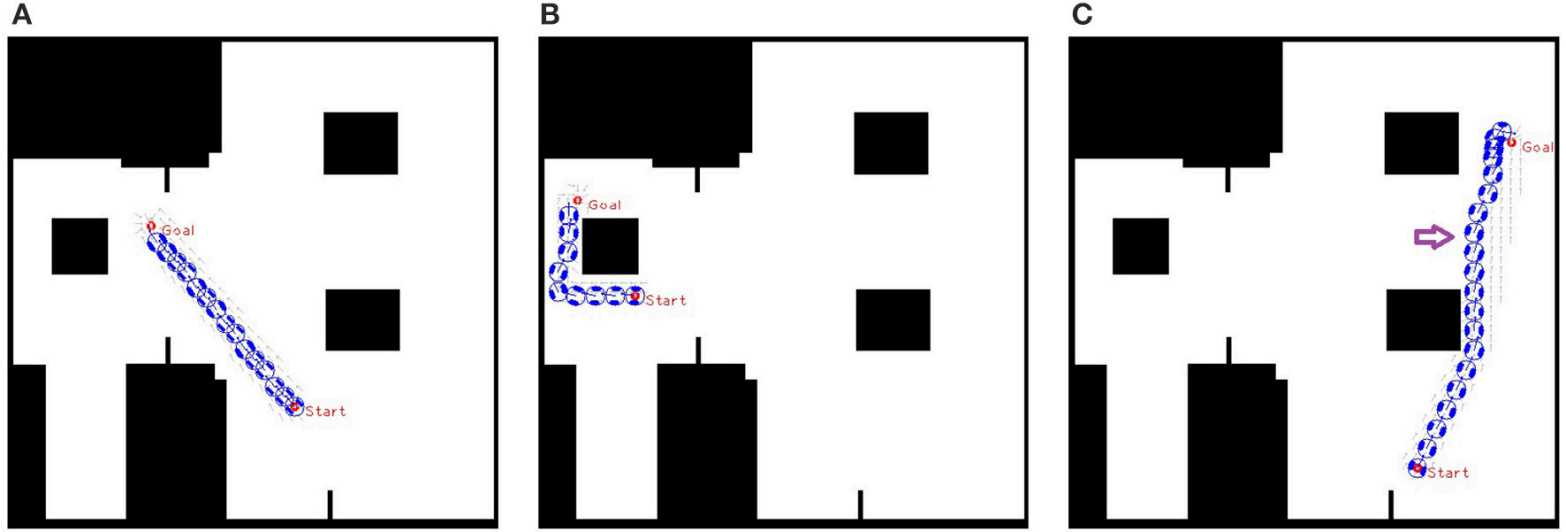
Agent moves from start to goal with static flow field where the window of static flow field is set as two times as the size of the agent. **(A,B)** an agent approaches the goal without the needs of re-estimating a new path, and **(C)** Theta^*^ is reactivated when the agent gets close to the second obstacle along its path (the location for activation is shown by the arrow symbol).

The aim of the static flow field is to convert the path found by Theta^*^ into a navigation field to avoid the needs of running Theta^*^ for every update of the agent position, and also to allow a more robust integration of the path planning with obstacle avoidance and velocity controls. Thus, only when the agent deviates from its designated path, due to slow adaptation to follow the navigation field, the path is required to be renewed using the Theta^*^ algorithm. Different examples of agent movements with static flow field are shown (Figure 6). In most situations, like examples given in Figures 6A,B, the agent approaches the goals without the needs of renewing the shortest path to the goal. However, in a particular case where the agent deviates from the designed path, Theta^*^ is reused to update the path to the goal (Figure 6C).

Different windows of the static flow field are evaluated. One hundred trials are attempted for each specific value of the window. In this experiment, a binary map of 50 × 50 m with a resolution of 10 pixels per meter is used. Each agent is presented by a bounding circle with a radius of 0.5 *m* and has the speed of 0.5*m*/*s* with *k*_ω_ = 1.2 (*k*_*u*_ is set to the speed of agents in all experiments). The influence distance *d*_0_ is set to 10 pixels (or one meter). For each trial, an agent moves from a starting point to a goal using only static flow field with velocity control. Pairs of starting and ending locations are selected randomly in the map. The results reveal that the bigger window is, the less number of running Theta^*^ the static flow field needs (Table 1). For the following experiments in sections 3.2 and 3.3, the window of two times of the agent size (*W* = 2*S*) is applied.

**Table 1:** Relationship between an average number of Theta^*^ used for static flow field to successfully drive an agent to its goal and window size (*W*).

**Window size**	***W* = *S*/4**	***W* = *S*/2**	***W* = *S***	***W* = 3*S*/2**	***W* = 2*S***	***W* = 5*S*/2**
						
Average number of Theta^*^	10.85	4.57	1.53	0.73	0.43	0.23

The size of an agent is S

### 3.2. Dipole flow field for crossing scenarios of two agents

**Figure 7:**
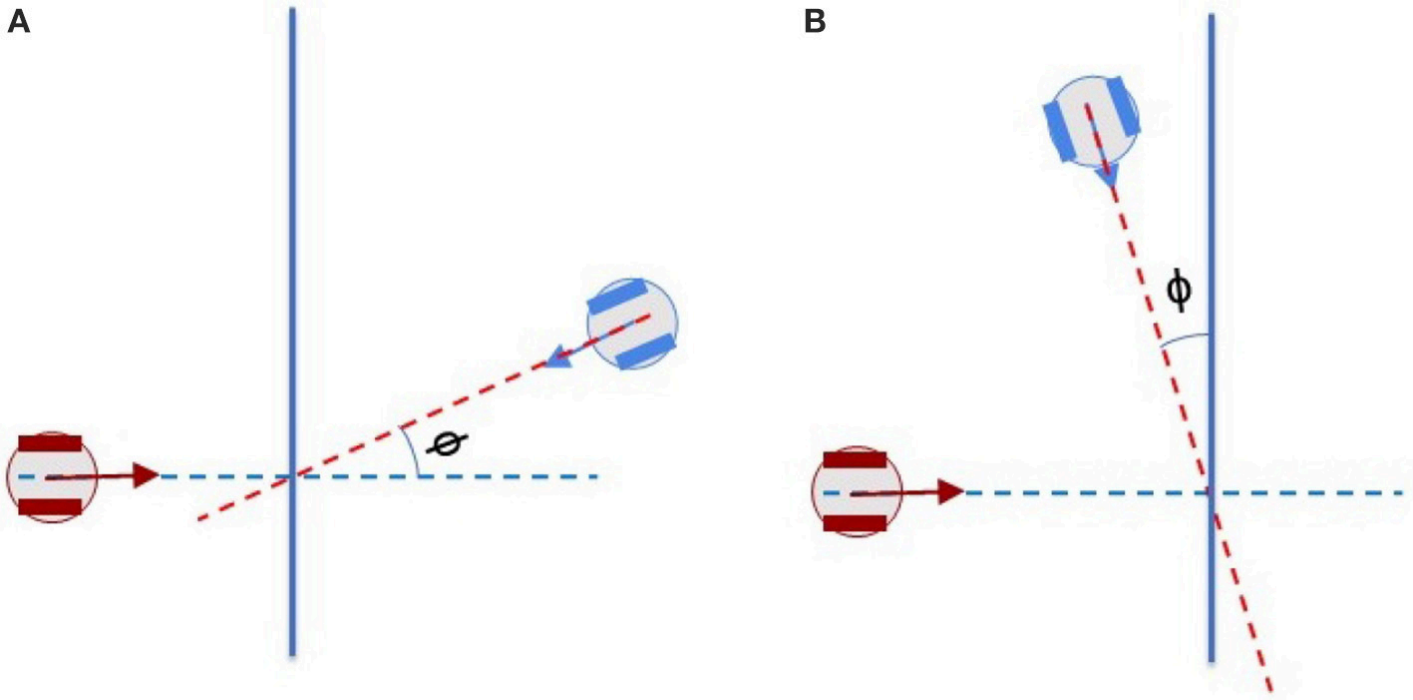
Crossing scenarios of two agents **(A)** Scenario 1: Two agents move toward each other with opposite directions and **(B)** Scenario 2: Two agents move toward each other with the heading angles of around 90°.

**Figure 8:**
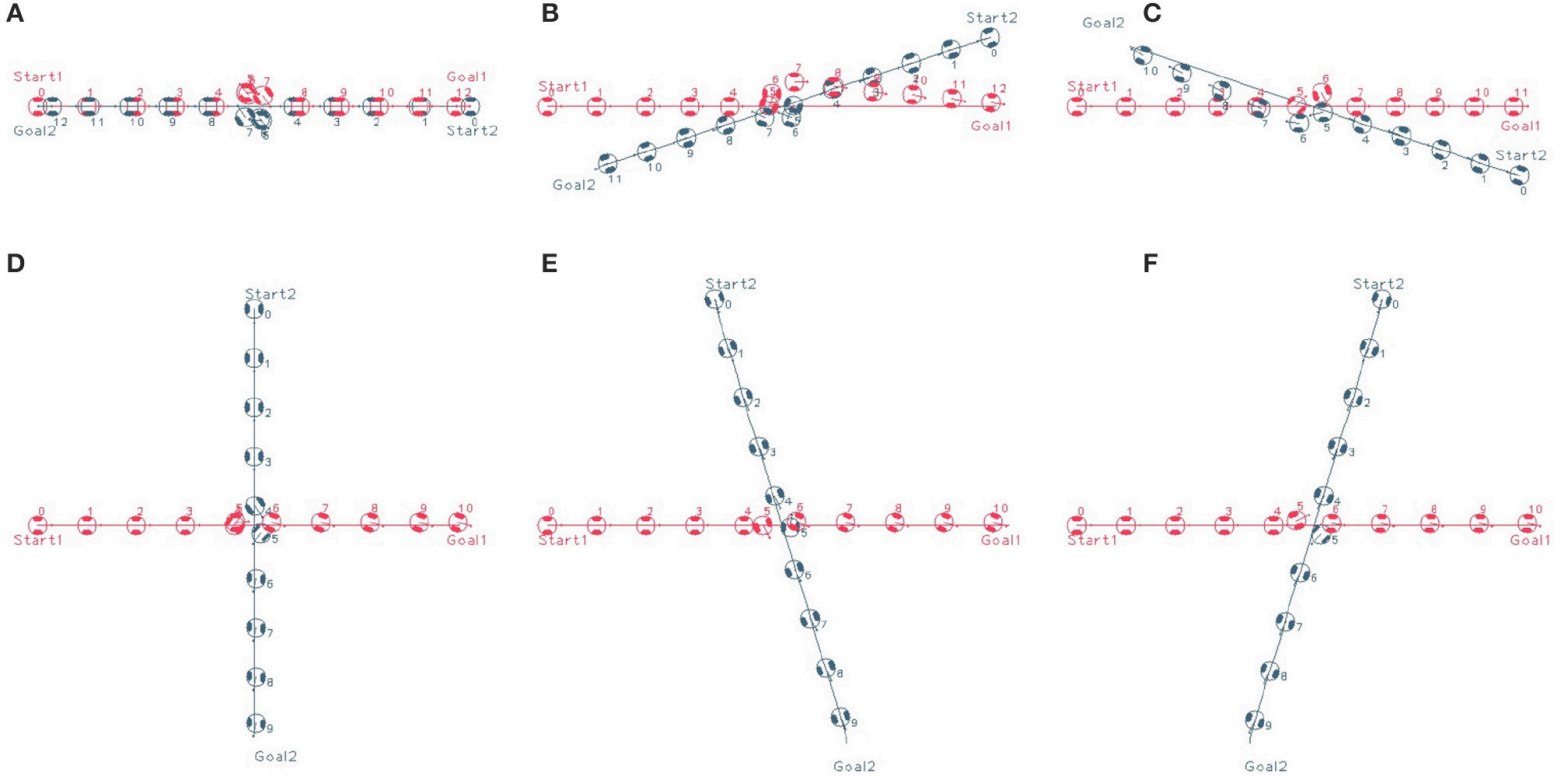
Trajectories of two agents in two scenarios. The first rows visualize the moving behaviors in dipole field of agents in Scenario 1 with different value of ϕ, **(A)** ϕ = 0, **(B)** ϕ > 0, and **(C)** ϕ < 0. Similarly, the second rows show the results of Scenario 2 with **(D)** ϕ = 0, **(E)** ϕ > 0, and **(F)** ϕ < 0. The time indices are used to show the location of agents at every 10 seconds.

**Figure 9:**
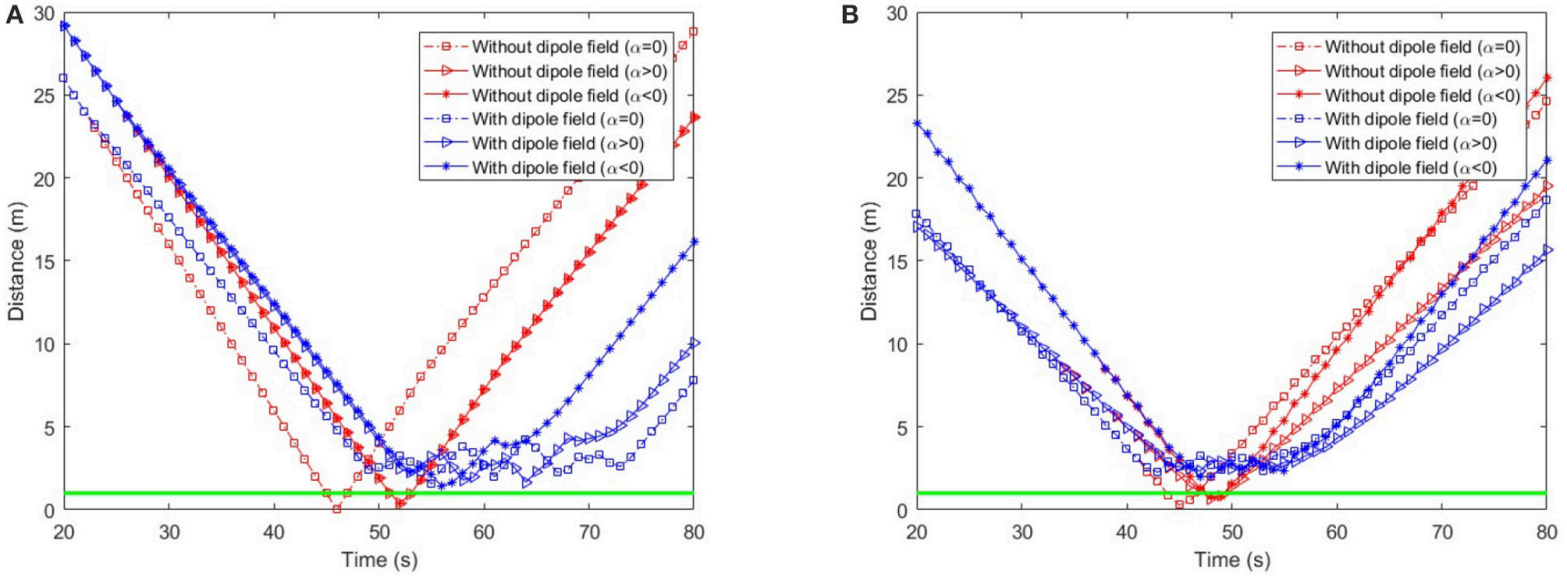
Distance of two agents over time in **(A)** Scenario 1 and **(B)** Scenario 2. The green baseline depicts the minimum distance between agents to avoid collisions.

To analyse the behavior of dipole flow field for obstacle avoidance, two simple scenarios, in which two agents are crossing each other are chosen (Figure 7). In Scenario 1, one of the agent moves from left to right and the other agent moves in the opposite direction. In Scenario 2, the first agent moves as previously, whereas the other agent starts in a position approximately 90^*o*^ to the first agent and moves from the left-hand to the right-hand side similar to the first agent. The variation of the moving directions of the two agents is also evaluated by validating different values of the heading angle of the second agent (ϕ = 0, ϕ > 0 and ϕ < 0, as seen in Figure 7). The size of the agents is set to a bounding circle with a radius of 0.5 *m* while the ratio β_*A*_/α = 5 and the coefficient γ = 1 are used. In both scenarios, the two agents moves at the same speed of 0.5*m*/*s* (with *k*_ω_ = 4) so that their path intersects in the middle of their way. However, with the help of repulsive forces generated by dipole field, the two agents are able to avoid the collisions (Figure 8). Besides, after a small deviation from the path, due to the dipole field interaction the agents turn back directly to their original paths to continue their routes toward their goals. The distance plots show that the minimum distance of two agents are remained above the agent's diameter (marked with the green line at 1.0 *m*, in Figure 9), thus there are no collisions present in the presented cases.

### 3.3. Dipole flow field for multi-agent and human-agent interaction

In the first part of this section, the behaviors of multiple agents within dipole-flow field are analysed. In the second part, the comprehensive evaluation of the dipole-flow field with the appearances of both agents and humans are preformed. Also, in the second part, the concluding experiment, which demonstrates the behavior of the agents in presence of human in a large and realistic area, is presented.

**Figure 10:**
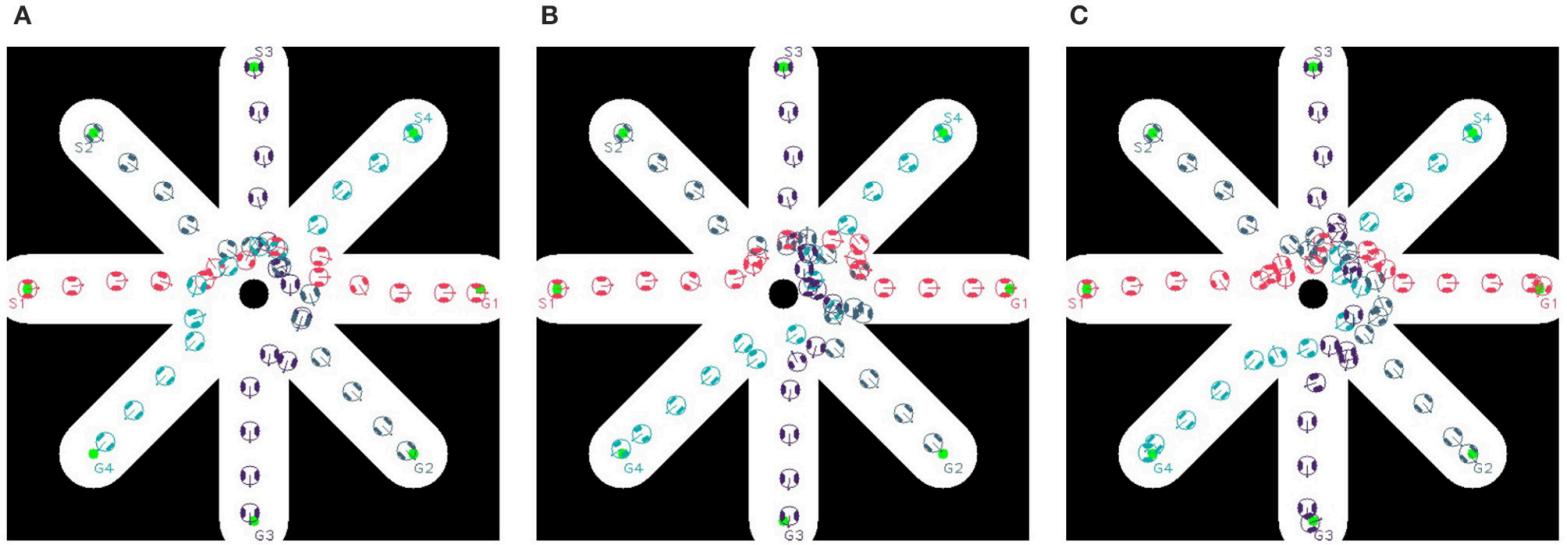
Trajectories of multiple agents moving **(A)** without dipole field, **(B)** with dipole field γ = 1, and **(C)** with dipole field γ = 0.95.

Four agents, positioned at different orientations with the same distance to the center of the map, take part in the first testing scenario (Figure 10). All agents are planned to cross the center, and move toward their goals symmetrical to their starting positions. The agents travel within a binary map of a size of 50 × 50 m with a resolution of 10 pixels per meter and with static obstacles so that the free-space of moving and avoiding other moving objects is limited. Also, the way to reach the goal is narrowed down and there is a traffic circle in the center of the map. Each agent has a radius of 0.5 *m* and a moving speed of 0.5*m*/*s* (with *k*_ω_ = 4). The quantitative measurement of obstacle avoidance (with β_*A*_/α = 5 and *d*_0_ = 25) is given by measuring the minimum distance among agents over time. The closest distance of two agents when they are moving if smaller than their size will reveal a collision between them.

As depicted in Figure 10A, using flow-field navigation all agents are able to reach their goals. However there are existing collisions between agents (1)-(4), (2)-(4), and (3)-(4) (**Figure 12A**) with regards to the agent's radius of 0.5 *m*. With dipole-flow-field navigation, agents show ability to avoid possible collisions. Finally, the control factor (γ) in dipole-flow field is evaluated to show its effects on the trajectories of agents in Figure 10C and the results in **Figure 12A**. When γ < 1 is used, the collisions are prevent in a better way by keeping the minimum distances among agents bigger. It is important to note that the trajectories of moving agents are visualized to not interfering with any static obstacles from the binary map.

**Figure 11:**
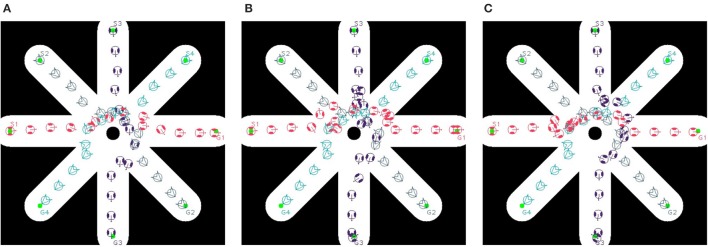
Trajectories of multiple agents moving and interacting with human **(A)** without dipole field, **(B)** with dipole field γ = 1, and **(C)** with dipole field γ = 0.95.

**Figure 12:**
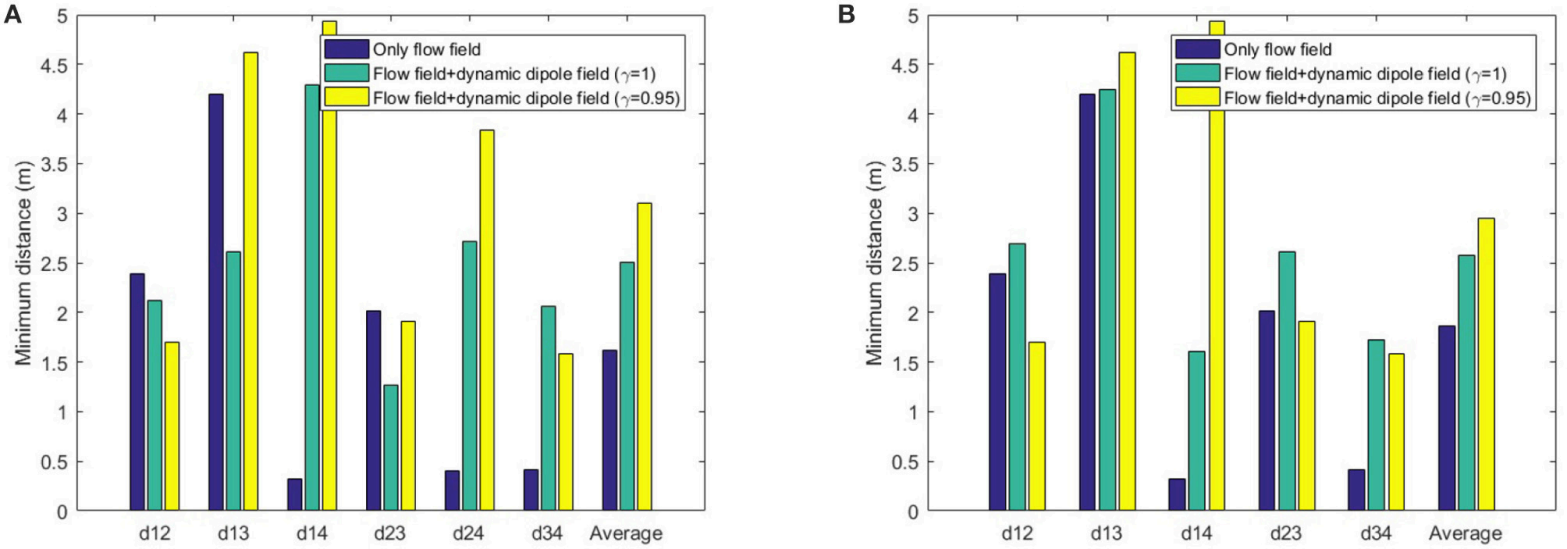
Minimum distance of agents over time in **(A)** multi-agent, and **(B)** human-agent interactions within dipole-flow field.

In order to evaluate dipole-flow-field for human-agent interaction, Agents 2 and 4 are replaced by two human subjects, which move as their agent counterparts, without caring the conflicts with agents. The moving trajectories of Agents 1 and 3 are described in Figure 11. Again, the collisions between agents are eliminated when agents are routed by the forces generated by dipole-flow field (Figure 12B).

**Figure 13:**
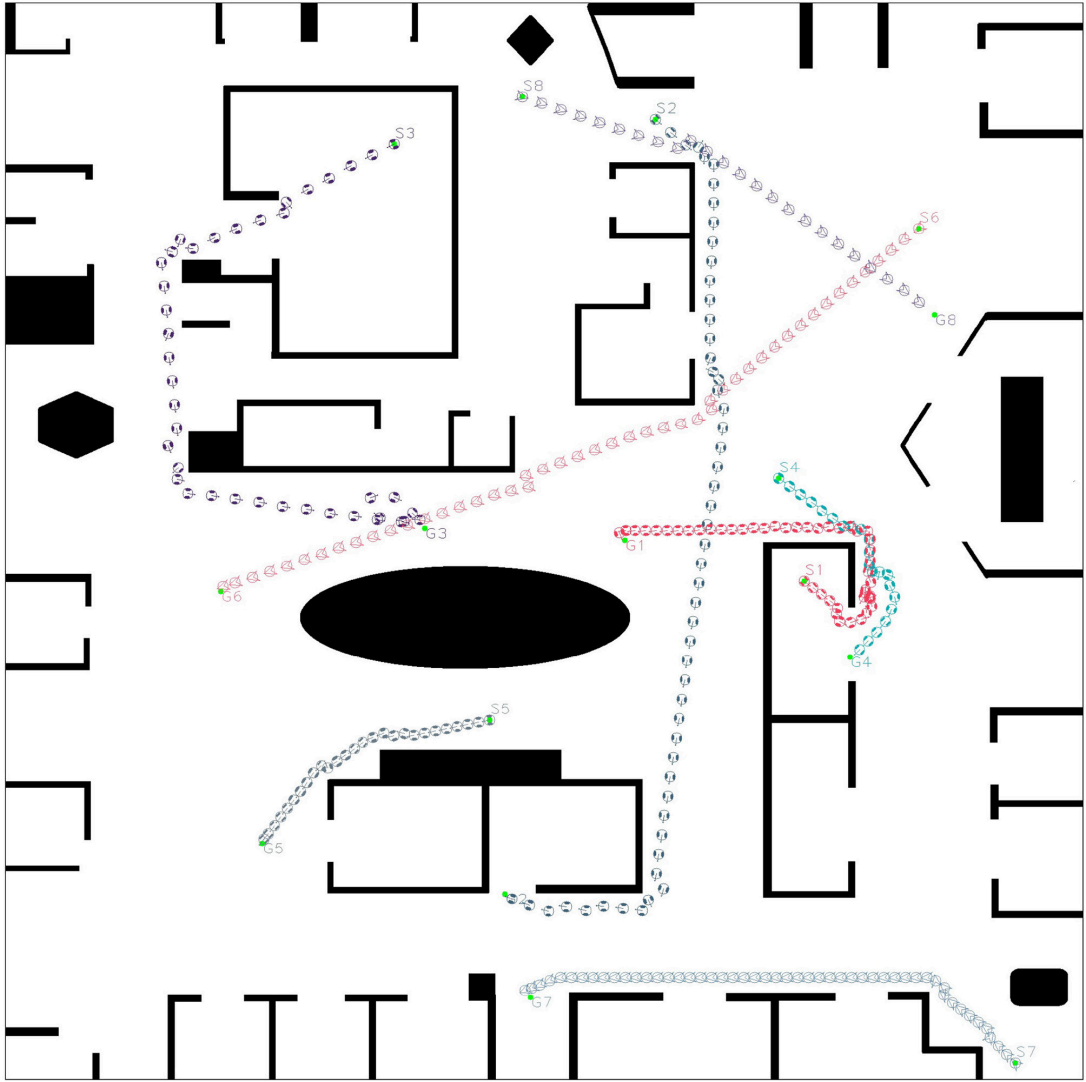
Dipole-flow field to control movements of multiple agents with the presence of three human in a 200 × 200 m large map which is visualized from a real working space. All agents are able to reach their goals with different speeds. While moving to goals, the two agents with indices 2 and 3 try to avoid the collision with human with index 6. Meanwhile, two agents with indices 1 and 4 also change directions to avoid collision with each other. In the case of the agent with index 3, the goal *G*3 is very close to the moving trajectory of human, therefore its way to the goal seems to be blocked until human with index 6 passes through *G*3. In consequence, the agent 3 must go back and later turn around to reach its goal. This behavior of moving is quite different with the scenario described in Figure 10B.

Finally, a general assessment of the dipole flow field for agent-agent and human-agent interactions within a large and complex binary map of variations of static obstacles drawn from a real building is presented. There are five moving agents and three human subjects in this evaluation. The map represents a part of the floor of a real building (width and length = 200 × 200 m with the same resolution of 10 pixels per meter). All agents have a bounding circle with a radius of 0.5 *m*, while β_*A*_/α = 50, *d*_0_ = 25, and γ = 0.95 are applied with bigger values than those of the previous experiments to help agents prevent collision from a further distance. An example of moving trajectories of different agents with human is shown in Figure 13. The experiments were repeated 100 times, and for each trial start and goal positions of both agents and humans were randomized. The requirement for finding the start position and goals was that the pairwise distances among them should be at least 2.0 *m*. The speed of the agents and human during the experiment is randomly assigned within a range of 0.5−1.5 *m*/*s* using a uniform distribution (with *k*_ω_ = 4). For each trial, agent-agent and human-agent the distances are recorded for the evaluation purposes. The overall result is summarized in Table 2.

**Table 2:** Evaluation of the minimum, and average, of agent-agent and human-agent distances over all 100 trials.

**Minimum of agent-agent distances (m)**	**Minimum of agent-human distances (m)**	**Average of minimum agent-agent distances (m)**	**Average of minimum agent-human distances (m)**
2.4	1.0	10.0	8.8

## 4. Conclusion and discussion

This paper has introduced a novel path planning algorithm for agents surrounded by static and multiple moving objects, including other robotic agents as well as humans subjects, all populating a realistic working space. The algorithm is able to process path planning in real-time by developing a navigation field so that the movements of agents is just simply controlled by the forces generated from this field. The attractive forces that drive the agents toward their desired goals are created by a static flow field. Simultaneously, the repulsive forces that prevent agent-agent, and human-agent, collisions are generated by a magnetic field of dipoles. The combination of the static flow field and dipole field forms a force to determine the moving directions of the agents at a specific time instance.

The evaluation of the proposed approach with the static flow field, dipole field and their combinations are conducted with distinctive experiments. With static flow field, it is obvious that an agent is able to move to its goals in a binary map of static obstacles with a minimum number of re-initializing the global path using the Theta^*^ algorithm. As can be seen in Table 1, the number of running Theta^*^ instances except the first initiation is less than one time in average (if the window effects of the static flow field is set to at least twice the size of the agents, *W* ≥ 2*S*). However, an unnecessary large window may cover otiose areas that affect the static flow field, leading to the trap of agent into a corner of the map. Therefore, the window size *W* = 2*S* is recommended to increase the robustness of the static flow field and to avoid the possibility of local traps.

Within the combined dipole-flow field, the robotic agents are well routed to their destinations, while possible collisions with other agents and human are taken into account. Regarding overall evaluation of the dipole-flow field to navigate agents in a complex scenario, the average minimum distance between any two agents remains at least double the radius of bounding circle, which indicates that there are no collisions (Table 2). The minimum human-agent distance is 1.0 *m*. However, such a recorded observation in which the human-agent distance is close to 1.0 *m* is only one case in 1, 500 obtained distance pairs (there is a group of five agents and three human subjects in the experiment so that the obtained pairs of human-agent is 1, 500 over 100 trials). Regarding the size of the human subjects, the bounding circle radius can be configured even less than 0.5 m, therefore it can be concluded that no occlusions happen in any of the simulation runs.

Recently, the aim of this work has moved toward holistic navigation solutions in real-world problems more specifically, mobile robots in densely populated areas such as, offices, and heavy vehicles in restricted spaces. Thus, by adding a control mechanism for the velocity, e.g., decreasing the speed to avoid possible collisions, as well as other measures will be investigated. Besides controlling the agents' velocity, the configuration of global paths with regards to multiple agents is also an important factor to ensure the reachability of all agents to their goals. In the current approach, only one optimal path to the goal is configured for each agent, without considering the conflicts with others. If any two agents enter into a very narrow area on opposite directions, as described by Kimmel and Berris (2016), the dipole forces mainly push them away to avoid collisions but not help them build new paths to their goals. Therefore, the two agents tend to follow the same planned paths again and again, leading to a deadlock situation. The proposed approach could be improved by setting multiple paths for each agent. Upon evaluating the location information of others and the binary map of environment, an agent is able to decide which path, even not optimal, it should follow to reach its goal. The aforementioned problem could be also addressed by exchanging information of planned paths among agents. All agents will negotiate to optimize the flow field on a global scale to avoid any deadlock situation. However, in this case the communication protocol will become more complicated and extra processing is needed at each agent side to optimize the global path with respect to the presence of other agents. Finally, the work will be extended with different classes of agents (Panagou, 2017), and with multiple heuristics of A^*^ (Aine et al., 2016) to allow more thoroughly investigation of the dependability factors, and constraints on the path planning problems. The intention is also to validate the algorithm using robots and humans in outdoor settings, that resemble the qualities of construction sites.

## Author contributions

This work is based on the original idea proposed by ME, later developed by LT. LT, and BC contributed to the design of experiments. All authors contributed to the development of system architecture and to the writing of the manuscript.

### Conflict of interest statement

The authors declare that the research was conducted in the absence of any commercial or financial relationships that could be construed as a potential conflict of interest.
